# Disparities in outcomes of patients admitted with diabetic foot infections

**DOI:** 10.1371/journal.pone.0211481

**Published:** 2019-02-04

**Authors:** Tze-Woei Tan, Chia-Ding Shih, Kirsten C. Concha-Moore, Muhanad M. Diri, Bo Hu, David Marrero, Wei Zhou, David G. Armstrong

**Affiliations:** 1 University of Arizona College of Medicine, Tucson, AZ, United States of America; 2 Southwest Academic Limb Salvage Alliance (SALSA), Los Angeles, CA, United States of America; 3 Keck School of Medicine at University of Southern California, Los Angeles, CA, United States of America; 4 Cleveland Clinic Foundation, Cleveland, OH, United States of America; Baylor College of Medicine, UNITED STATES

## Abstract

**Objective:**

The purpose of this study was to evaluate the disparities in the outcomes of White, African American (AA) and non-AA minority (Hispanics and Native Americans (NA)), patients admitted in the hospitals with diabetic foot infections (DFIs).

**Research design and methods:**

The HCUP-Nationwide Inpatient Sample (2002 to 2015) was queried to identify patients who were admitted to the hospital for management of DFI using ICD-9 codes. Outcomes evaluated included minor and major amputations, open or endovascular revascularization, and hospital length of stay (LOS). Incidence for amputation and open or endovascular revascularization were evaluated over the study period. Multivariable regression analyses were performed to assess the association between race/ethnicity and outcomes.

**Results:**

There were 150,701 admissions for DFI, including 98,361 Whites, 24,583 AAs, 24,472 Hispanics, and 1,654 Native Americans (NAs) in the study cohort. Overall, 45,278 (30%) underwent a minor amputation, 9,039 (6%) underwent a major amputation, 3,151 underwent an open bypass, and 8,689 had an endovascular procedure. There was a decreasing incidence in major amputations and an increasing incidence of minor amputations over the study period (P < .05). The risks for major amputation were significantly higher (all p<0.05) for AA (OR 1.4, 95%CI 1.4,1.5), Hispanic (OR 1.3, 95%CI 1.3,1.4), and NA (OR 1.5, 95%CI 1.2,1.8) patients with DFIs compared to White patients. Hispanics (OR 1.3, 95%CI 1.2,1.5) and AAs (OR 1.2, 95%CI 1.1,1.4) were more likely to receive endovascular intervention or open bypass than Whites (all p<0.05). NA patients with DFI were less likely to receive a revascularization procedure (OR 0.6, 95%CI 0.3, 0.9, p = 0.03) than Whites. The mean hospital length of stay (LOS) was significantly longer for AAs (9.2 days) and Hispanics (8.6 days) with DFIs compared to Whites (8.1 days, p<0.001).

**Conclusion:**

Despite a consistent incidence reduction of amputation over the past decade, racial and ethnic minorities including African American, Hispanic, and Native American patients admitted to hospitals with DFIs have a consistently significantly higher risk of major amputation and longer hospital length of stay than their White counterparts. Native Americans were less likely to receive revascularization procedures compared to other minorities despite exhibiting an elevated risk of an amputation. Further study is required to address and limit racial and ethnic disparities and to further promote equity in the treatment and outcomes of these at-risk patients.

## Introduction

Foot ulceration or infection is a significant lower extremity complication affecting people with diabetes. It is estimated up to 34% of adults with diabetes will develop a diabetic foot problem over their lifetime.[1, 2] Diabetic foot ulcerations (DFUs) are associated with increased risk of infection, lower extremity amputation, and death.[3–5] Serving as a portal of entry for microorganisms, more than one-third of DFUs have an infection on presentation.[6] Patients with diabetic foot infections (DFIs) have poor clinical outcomes.[7] More than 15% of patients with DFIs will die within one year of diagnosis and 17% will undergo major amputation.[7]

Racial and ethnic minorities experience disparities in both rates and treatment of lower amputation.[8–11] The prevalence of diabetic-related amputation is 2–3 times greater for African Americans than non-Hispanic Whites.[12] Race has been demonstrated to an independent and significant risk factor for amputation in peripheral arterial disease (PAD) and diabetes.[8, 13, 14] Minorities have a more advanced clinical presentation and have more comorbidities.[14] African Americans are more likely to undergo primary amputation than revascularization to improve arterial blood flow.[14–17] A majority of studies have focused on Whites and AAs. Consequently, disparities among racially and ethnically diverse patients with DFIs are not well studied.[18, 19]

We utilized the National Inpatient Sample (NIS) to examine the outcomes of patients admitted to the hospital for DFIs across various racial/ethnic groups with emphasis on non-AA minorities including Hispanics and Native Americans (NAs). We also aimed to better understand the influence of race/ethnicity on endovascular procedures and open bypass surgeries performed for revascularization. We hypothesized that there would be significant disparities in the outcome and management of patients with DFIs based on ethnicity.

## Materials and methods

### Study cohort

The NIS was retrospectively queried to identify all the patients who were admitted to hospitals with DFIs from 2003 to 2015. The NIS, developed as part of the Healthcare Cost and Utilization Project (HCUP), is the largest publicly available all-payer health care database and collects data for more than 7 million hospital stays every year.[20] It is a stratified sample of approximately 20% of discharges from academic and community US hospitals, excluding federal hospitals such as the Veterans Affairs Hospitals and Indian Health Service Hospitals.[20] The NIS contains clinical data and resource-use information including hospital characteristics and expected payment source for each hospital stay.[20] The Institutional Review Board at the University of Arizona College of Medicine approved the use of the de-identified data for this study and informed consent was waived.

The International Classification of Disease, Ninth Revision, Clinical Modification (ICD-9 CM) codes were used to identify adult patients (≥ 18 years old) with DFIs using the modified Sohn et al. methodology.[21] Discharges with ICD-9 codes of diabetes (250.xx) and one of the following ICD-9 codes: gas gangrene (040.0), osteomyelitis (730.07, 730.17, 730.27, 730.97), cellulitis or abscess of toe (681.1), or cellulitis of and abscess of foot, except toes (682.7), with or without ulcer (440.23, 707.10, 707.14, 707.15) were included in the study cohort. Patients with PAD were identified using ICD-9 codes 250.70, 250.71, 443.0, and 459.81. Patients < 18 years old and with missing race/ethnicity information were excluded.

### Outcomes and variables

We categorized patients based on the race and ethnicity information in the NIS which includes White, African American (AA), Hispanic, Native American (NA), and other. Demographics, medical comorbidities, hospital characteristics, and insurance information were also evaluated. The definitions of the medical comorbidities and hospital characteristics are described in detail on the website.[20] In the NIS the severity of the comorbidities was classified into four classes based on the All Patient Refined-DRGs (APR-DRGs).[20]

The primary outcome measures were major and minor lower extremity amputations which were identified using ICD-9 procedure codes. Major amputation was defined as a lower extremity amputation above the ankle and minor amputation as an amputation below the ankle. Other outcomes included were in-hospital mortality, hospital length of stay (LOS), endovascular intervention (ICD-9 code 39.5, 39.9), and open infrainguinal bypass surgery (ICD-9 code 39.25, 39.29). The incidence of hospital admission, minor amputation, major amputation, endovascular intervention, and open surgery were studied over the period (2003 to 2015) among the racial groups.

### Statistical analyses

The means and standard deviations were used to describe continuous characteristics whereas counts and percentages were used for categorical characteristics. Patient outcomes including mortality, major and minor amputations, and endovascular/open revascularization procedures, were estimated as proportions by year and racial group. The Chi-squared test was used to compare the categorical outcomes among the racial groups. Analysis of variance (ANOVA) was used to compare the continuous outcomes such as LOS among the racial groups.

Multivariable logistic models were performed to study the association between racial and ethnic groups and outcomes, including major and minor amputations, endovascular intervention, and open surgery. Factors that were significant in univariate analyses were incorporated into the multivariable models, which included age, gender, obesity, renal failure, peripheral arterial disease (PAD), APR-DRG severity class, and health insurance. Odds ratio (OR) with a 95% confidence interval (CI) was used to describe the association between racial and ethnic groups and outcomes. A two-sided p-value less than 0.05 was considered to be statistically significant in this study. All analyses were performed with R-studio (Boston, MA).

## Results

During the study period between 2003 to 2015, there were 150,701 admissions for DFIs, including 98,361 Whites (65%), 24,583 AAs (16%), 24,472 Hispanics (16%), 1,631 NAs (1.1%), and 1,654 identified as “other.” The mean age was 59 ± 13.7 years, 66.6% were male, and approximately 31% had a history of PAD (Table 1). Sixty-three percent had either Medicaid or Medicare insurance, 46% were admitted to a teaching hospital, and 89% were treated in an urban hospital. The mortality rate was 0.7%. In the study cohort 9,039 (6%) and 45,278 (30%) underwent major and minor amputations, respectively (Table 2). Approximately, 6% (8,689) underwent endovascular procedures and 2% (3,151) received open revascularizations.

**Table 1:** Demographic for patients with diabetic foot infections.

Characteristic	Overall N = 150,701	White N = 98,361	African American N = 24,583	Hispanic N = 24,472	Native American N = 1,631	Other N = 1,654
Age, mean (SD), years	59.2 (13.7)	60.7 (13.4)	55.6 (14.1)	57.1 (13.4)	54.4 (13.3)	61.3 (14.2)
Female gender, n (%)	50,274 (33.4)	32,261 (32.8)	9,362 (38.1)	7,463 (30.5)	599 (36.7)	589 (35.6)
Obesity, n (%)	27,364 (18.3)	19,724 (20.2)	3,964 (16.2)	3,197 (13.1)	281 (17.2)	198 (12)
Hypertension, n (%)	104,626 (69.9)	66,747 (68.5)	18,631 (76.2)	16,865 (69)	1,188 (72.8)	1,195 (72.3)
Congestive heart failure, n (%)	29,902 (14)	14,902 (15.3)	3,302 (13.5)	2,333 (9.5)	138 (8.5)	227 (13.7)
Chronic lung disease, n (%)	18,131 (12.1)	13,786 (14.1)	2,365 (9.7)	1,672 (6.8)	162 (9.9)	146 (8.8)
Peripheral artery disease, n (%)	46,212 (30.9)	29,292 (30%)	7,531 (30.8)	8,380 (34.3)	450 (27.6)	559 (33.8)
APRDRG Risk, %.						
1	46,464 (31)	29,416 (30.2).	7,525 (30.8)	8,528 (34.9)	556 (34.1)	439 (26.6)
2	70,382 (47)	46,966 (48.2)	11,098 (45.4)	10,810 (44.2)	783 (48)	725 (43.9)
3	28,887	18,534 (19)	5,150 (21.1)	4,506 (18.4)	266 (16.3)	431 (26.1)
4	(19.3) 3,921 (2.6)	2,573 (2.6)	669 (2.7)	595 (2.4)	26 (1.6)	58 (3.5)
Insurance, n (%)						
Medicare/Medicaid	95,338 (63.3)	62,161 (63.2)	15,806 (64.3)	15,244 (62.3)	1,000 (61.3)	1,127 (68.1)
Private	37,536 (24.9)	26,716 (27.2)	5,523 (22.5)	4,596 (18.8)	338 (20.7)	363 (21.9)
Other	17,827 (11.8	9,484 (9.6)	3,254 (13.2)	4,632 (18.9)	293 (18)	164 (9.9)
Hospital setting, %						
Urban	134,015 (89.2)	85,013 (86.7)	22,585 (93.3)	23,372 (95.7)	1,210 (75)	1,562 (94.7)
Teaching	69,080 (46)	40,977 (41.8)	14,509 (59.3)	12,138 (49.7)	621 (38.5)	835 (50.6)
Hospital size, %						
Small	22,067 (14.7)	15,494 (15.8)	3,099 (12.7)	2,968 (12.2)	267 (16.6)	239 (14.5)
Medium	41,819 (27.8)	26,938 (27.5)	6,971 (28.5)	7,040 (28.8)	424 (26.3)	446 (27)
Large	86,337 (57.5)	55,617 (56.7)	14,417 (58.9)	14,417 (59)	922 (57.2)	964 (58.5)
Region, %						
Northeast.	33,645 (22.3)	23,931 (24.3)	5,760 (23.4)	3,584 (14.6)	111 (6.8)	259 (15.7).
Midwest.	22,275 (14.8)	17,513 (17.8)	3,371 (13.7)	1,004 (4.1)	286 (17.5)	101 (6.1).
South.	65,577 (43.5)	41,140 (41.8)	13,445 (54.7)	10,280 (42)	415 (25.4)	297 (18)
West	29,204 (19.4)	15,777 (16)	2,007 (8.2)	9,604 (39.2)	819 (50.2)	997 (60.3)

**Table 2:** Outcomes of patients with diabetic foot infections.

Characteristic	Overall N = 150,701	White N = 98,361	African American N = 24,583	Hispanic N = 24,472	Native American N = 1,631	Other N = 1,654
Mortality, n (%)	1,047 (0.7)	690 (0.7)	178 (0.7)	158 (0.6)	8 (0.5)	13 (0.8)
Minor amputation, n (%)	45,278 (30)	28,806 (29.3)	7,774 (31.6)	7,719 (31.5)	453 (27.8)	526 (31.8)
Major amputation, n (%)	9.039 (6)	5,268 (5.4)	1,861 (7.6)	1,678 (6.9)	116 (7.1)	116 (7)
Open bypass, n (%)	3,151 (2.1)	1,992 (2)	524 (2.1)	575 (2.3)	14 (0.9)	46 (2.8)
Endovascular intervention, n (%)	8,689 (5.8)	5,407 (5.5)	1,443 (5.9)	1,650 (6.7)	81 (5)	108 (6.5)

**Fig 1:**
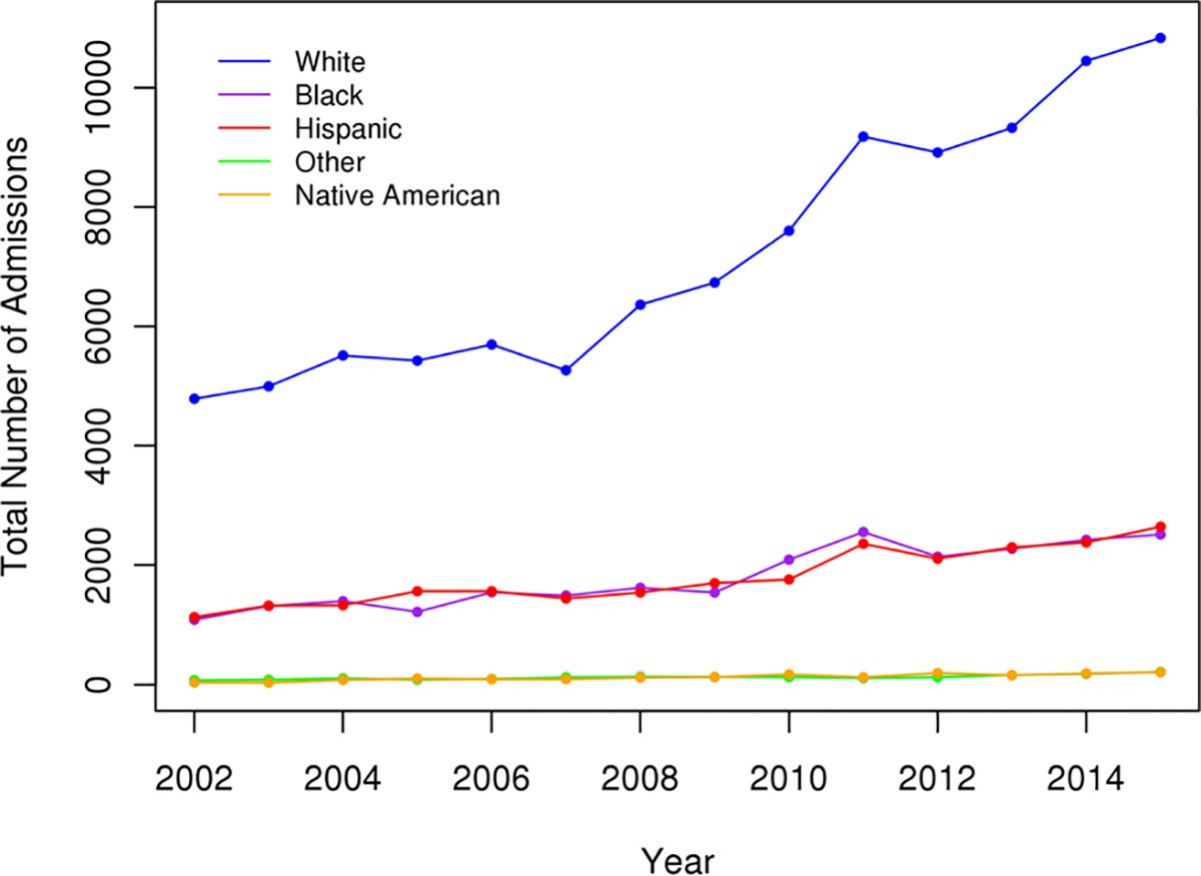
Inpatient admissions for diabetic foot infections.

**Fig 2:**
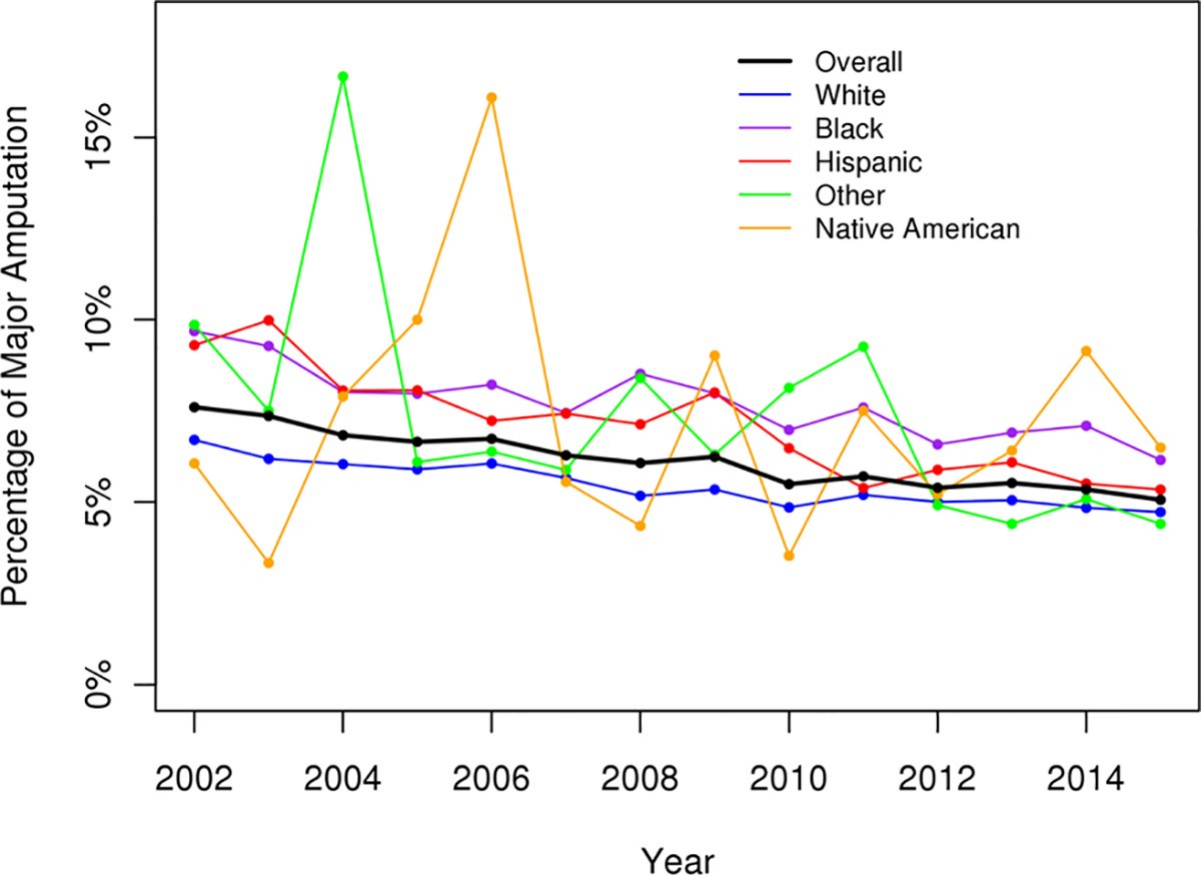
Percentage of major amputations for diabetic foot infections (overall p < .05).

**Fig 3:**
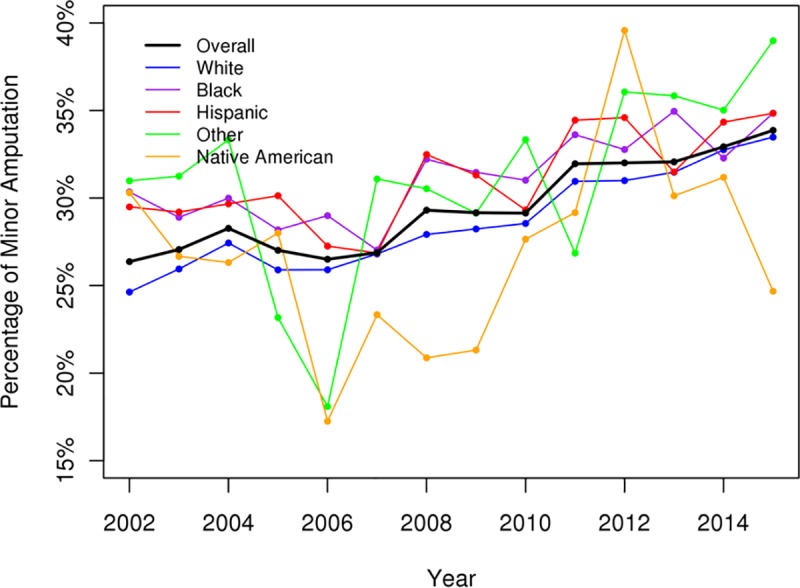
Percentage of minor amputation for diabetic foot infection (overall p < .05).

**Fig 4:**
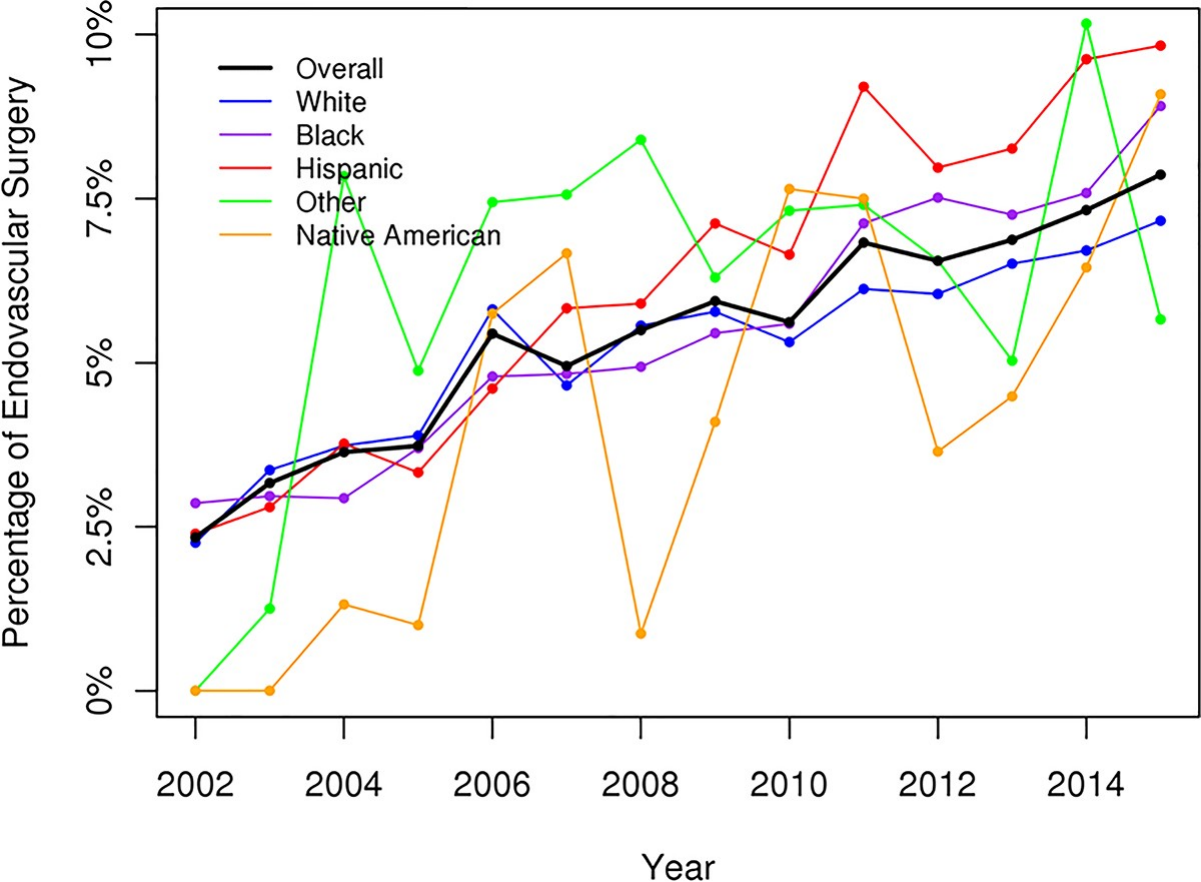
Endovascular procedure for diabetic foot infection (overall p < .05).

**Fig 5:**
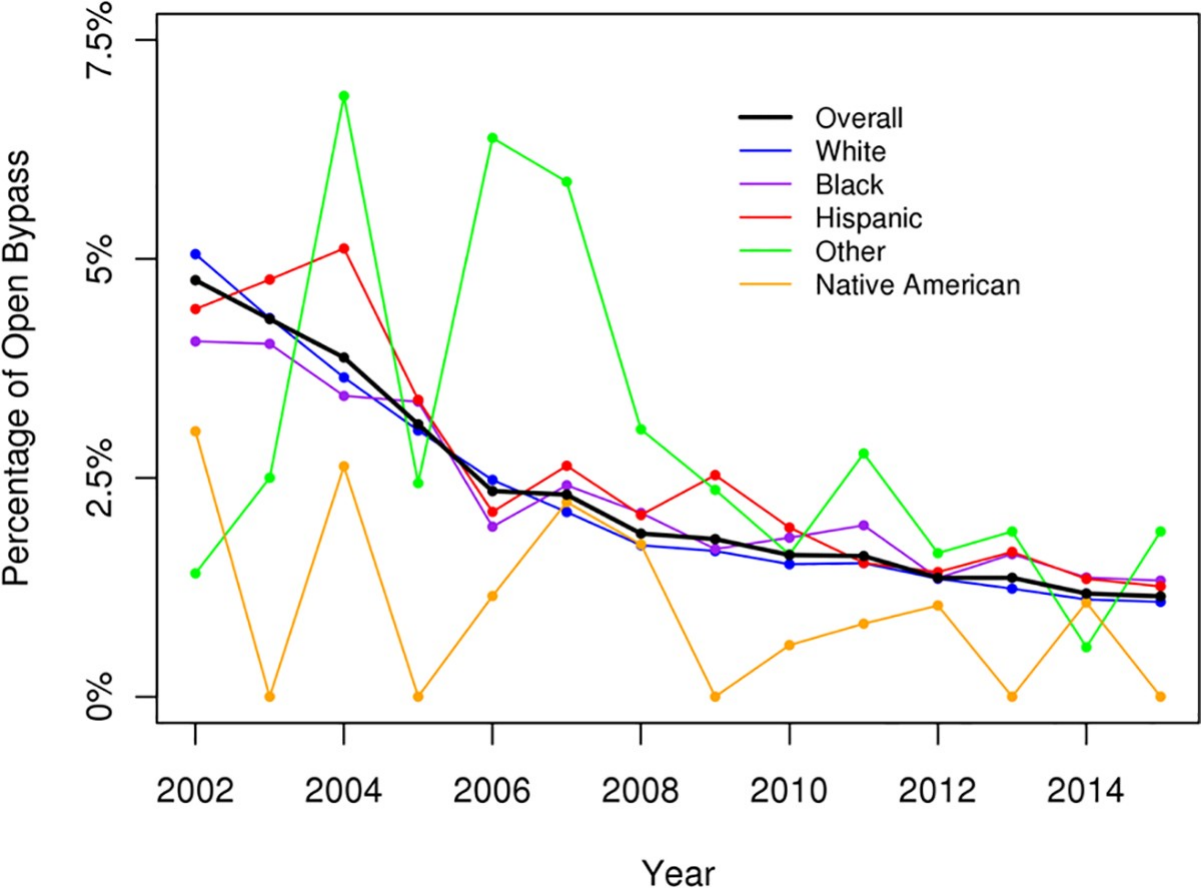
Open bypass for diabetic foot infection (overall p < .05).

The overall numbers of admission for DFIs had increased over the study period and were greatest among White, AA, and Hispanic patients (Fig 1). There was a significant decrease in the incidence of major amputations (p < .05) and an increase in the incidence of minor amputations for DFIs over the study period (p < .05) (Figs 2 and 3). All racial groups, except NAs, demonstrated a significant decreasing incidence of major amputations (Fig 2). NA patients with DFIs had an increasing incidence of major amputations over the study period. The annual percentage of endovascular procedures increased whereas there was a decreasing incidence in open revascularization surgery between 2003 to 2015 (p < .05) (Figs 4 and 5).

In bivariate analysis, although the mortality was similar among all groups, major amputations were significantly higher for AAs (7.6%), Hispanics (6.9%) and NAs (7.1%) than the Whites (5.4%, p < .001) (Table 2). The number of minor amputations were higher for AAs (31.6%) and Hispanics (31.5%) than Whites (29.3%) and NAs (27.8%, p < .001). NAs (0.9%) received the lowest rate of open bypass compared to Whites (2%), AAs (2.1%), and Hispanics (2.3%, p < .001) with DFIs. Endovascular procedures were highest among AAs (5.9%) and Hispanics (6.7%), and lowest among Whites (5.5%) and NAs (5%, p < .001).

In multivariate analysis, risk of major amputation was higher for NAs (OR 1.47, 95% CI 1.2, 1.8, p < .001), AAs (OR 1.44, 95% CI 1.36, 1.53, p < .001), and Hispanics (OR 1.33, 95% CI 1.25, 1.41, p < .001), when comparison was made to Whites with DFIs. Other risk factors for major amputation were male gender (OR 1.19, 95% CI 1.13, 1.25, p < .001), peripheral artery disease (OR 1.2, 95% CI 1.3, 1.5, p < .05), renal failure (OR 1.08, 95% CI 1.02, 1.13, p = .004), APRDRG class 2 (OR 1.61, 95% CI 1.5, 1.72, p < .001), class 3 (OR 4.21, 95% CI 3.91, 4.53, p < .001), and class 4 (OR 8.60, 95% CI 7.77, 9.51, p < .001). Obesity (OR 0.78, 95% CI 0.73, 0.83, p < .001) and commercial health insurance (OR 0.81, 95% 0.76, 0.85, p < .001) were associated with lower risk of a major amputation.

The risk of minor amputation was significantly higher for AAs (OR 1.14, 95% CI 1.11, 1.18, p < .001) and Hispanics (OR 1.10, 95% CI 1.07, 1.14, p < .001). Compared to Whites with DFIs, AAs (OR 1.23, 95% CI 1.12, 1.36, p < .001) and Hispanics (OR 1.34, 95% CI 1.22, 1.48, p < .001) were more likely to undergo open bypass whereas NAs were less likely to have open bypass surgery (OR 0.56, 95% CI 0.33, 0.95, p = .03). Hispanics (OR 1.41, 95% CI 1.33, 1.50, p < .001) and AAs (OR 1.20, 95% CI 1.12, 1.27, p < .001) were also more likely to undergo endovascular procedures when the comparison was made to Whites with DFIs.

The overall LOS was 8.3 ± 7.7 days (Table 3) and was longer when a minor amputation (9.5 ± 7.5 days) or a major amputation (14.9 ± 12.3 days) was performed. The hospital LOS for overall patients with DFIs, those without any amputation, those with a minor amputation, and patients undergoing major amputations were significantly longer for AAs and Hispanics when comparison was made with Whites (Table 3).

**Table 3:** Hospital length of stay for patients with diabetic foot infections.

Length of Stay	Overall N = 150,701	White N = 98,361	African American N = 24,583	Hispanic N = 24,472	Native American N = 1,631	Other N = 1,654
All DFI, days	8.3 ± 7.7	7.9 ± 7.3	9.2 ± 8.4	8.6 ± 8.0	7.8 ± 6.5	9.7 ± 10.0
DFI with no minor or major amputation	7.1 ± 6.7	6.9 ± 6.4	7.8 ± 7.4	7.3 ± 7.1	6.7 ± 5.7	8.1 ± 8.6
DFI with minor amputation	9.5 ± 7.5	9.1 ± 7.1	10.5 ± 8.0	9.7 ± 8.0	8.9 ± 6.4	11.4 ± 11.3
DFI with major amputation	14.9 ± 12.3	14.5 ± 12.7	16.0 ± 11.9	15.0 ± 11.8	13.6 ± 9.6	16.3 ± 11.6

## Discussion

In our study using the most recently available data from the NIS, the percentages of major amputations among patients admitted for DFIs had decreased significantly from 2003 to 2015. The decreasing incidence of major amputations were observed in all racial/ethnic groups except the NA group, which showed increasing rates of major amputations over time. Our study confirmed the increased risks of lower extremity amputation among AAs and non-AA minorities compared to Whites. Native Americans with DFIs had highest risk of amputations, follow by AAs and Hispanics. Other risk factors associated with amputation were male gender, PAD, renal failure, and medical comorbidities. AAs and Hispanics with DFIs exhibited higher risks of amputation and were more likely to require revascularization procedures during hospitalization suggesting a role of early diagnosis and aggressive management of PAD and diabetic foot ulceration before infection among these at-risk groups.

Our findings are consistent with other recent studies demonstrating an overall reduction in diabetes-related lower extremity amputations in the US and across the world.[22–24] The decline was observed across all racial groups including Whites, AAs, and Hispanics.[22, 23] During the same time there were increased percentages of minor amputations, which might reflect the increasing use of aggressive toe and foot amputations to control the foot infection, resulting in improved limb salvage. This rising trend in minor amputations over the study period was observed across Whites and among the minority groups. One possible reason for the reduction in major amputations might be the increase in the revascularization procedures among patients with DFIs.[25] Peripheral artery disease is one of the major risk factors for amputation in patients with diabetic foot ulcerations and revascularization procedures have been shown to be effective for reducing risk for amputation and wound healing.[26, 27] Consistent with trends for PAD treatment across the US, there was an increase in endovascular interventions and a decrease in open bypass surgery in this study.[28] Further studies will be required to evaluate the relationship between the increase in endovascular procedures and the decrease in the rate of amputations.

Racial and ethnic groups experience disparate rates of diabetic foot problems and amputations.[12, 18, 29, 30] Our data demonstrated the increased rates of major and minor amputations in AAs and Hispanics with DFIs. After controlling for medical comorbidities including PAD and renal failure, the risk of undergoing major amputation remained elevated and was 33%, 44%, and 47% higher for Hispanics, AAs, and NAs respectively compared to their White counterparts. This observation may reflect that racial and ethnic minorities, especially AA patients, are more likely to present late in the course of their disease compared to White patients.[15, 18, 29] Significant numbers of minority patients had osteomyelitis or gangrene and had more extensive cardiovascular risk factors on presentation.[13, 15, 18] In addition, socioeconomic status such as insurance status and income level might be responsible for the disparate risks of amputation in minority patients.[8, 13, 15, 31, 32] When the comparison was made with White patients, non-White patients are more likely to have low income or live in a low-income area and have suboptimal medical insurance.[13, 31, 33] Similarly, minority patients with DFIs including AAs, Hispanics, and NAs were noted to be less likely to have a commercial insurance and more likely to have Medicaid or Medicare in this study.

Of particular concern is the fact that AAs and Hispanics with PAD are more likely to undergo primary amputation without any attempt at revascularization.[9, 15–17, 30, 34, 35] Minority patients are 2–3 times less likely to receive any endovascular procedure or open bypass for PAD than Whites, which might suggest reduced access to medical care and revascularization procedures.[15, 30, 34] Our findings, demonstrating a significantly higher rate of revascularization procedures in minority patients than compared to White patients during hospital admission, might similarly suggest issues with access to medical care among minority patients. Minority patients are more likely to have an advanced presentation of DFI and tend to utilize the hospital as the primary avenue to receive medical care for their diabetic foot problems.

It is worthwhile to emphasize the findings of the NA patients in this study. Native Americans with DFIs had the highest risk of major amputation among all the racial groups and were the only group which exhibited an increasing incidence of major amputations over the study period. Other groups including the Whites, AAs, and Hispanics with DFIs showed a reduction in major amputation over time. Although other minority groups underwent higher numbers of endovascular procedures and open bypass than White patients, the NA patients were less likely to receive any revascularization procedure during their hospitalization. In addition, NAs were more likely to be admitted to rural hospitals and non-teaching hospitals for treatment of DFIs. It is possible that NAs with DFIs presented to the hospital later than the AAs or Hispanics and limb salvage is not possible due to the extent of the foot infection. Other possible explanations might be that the type of hospital that NAs received care for DFIs may not be equipped to offer complex limb salvage procedures. The influence of race on provider bias and preference is well described in modern day medicine and might be responsible for the disparities of revascularization procedure among the NAs observed in this study.[36, 37]

Our study has important limitations. First, this is an observational study using administrative records to compare the outcomes of patients with DFIs. Although we tried to control for potential confounders using multivariable analyses, there are potential risk factors and selection bias not measured or accounted for in the analyses. Second, our analyses are limited by in-hospital outcomes due to the limitation of the NIS. The medium and long-term outcomes are likely important to be taken into consideration to properly evaluate the disparities of patients with DFIs. Third, we do not know the severity and extent of the foot infection for individual patient to adequately adjust for the risk of major amputation. This study is limited by the used of ICD-9 codes to identify our study cohort which can be misleading due to the risk of over or under diagnosing. Fourth, we do not know the exact reason why the decision of amputation versus revascularization procedure was made for the given patient. Finally, patients who were admitted to Indian Health Service Hospitals and Veteran Affairs Medical Centers were not included due to limitation of the NIS. Even with the limitations mentioned above, we were able to use the NIS to evaluate a large cohort of patients who were admitted for DFIs. Although this is not a prospective study, our findings reflect the actual real-world outcomes of patients with DFIs across various hospital settings, including community and academic hospitals, rural and urban hospitals, and teaching and non-teaching hospitals. In this context, there is clear evidence that race/ethnic disparities persist in the treatment of DFIs.

In conclusion, although the rate of major amputations has decreased over time, our findings suggest racial and ethnic disparities among patients admitted to hospital with DFIs persist. Native Americans were less likely to receive any revascularization procedure compared to other minorities despite exhibiting the highest risk of amputation. African Americans and Hispanics had a higher risk of major amputation and were more likely to undergo any revascularization procedure, likely due to delay in presentation and management of their diabetic foot problems. Further study is required to address and limit racial and ethnic disparities in the treatment and outcomes of these at-risk patients and to further promote equity in care.

## Supporting information

S1 Data (CSV)
