# Correction: Disparities in outcomes of patients admitted with diabetic foot infections

**DOI:** 10.1371/journal.pone.0215532

**Published:** 2019-04-11

**Authors:** Tze-Woei Tan, Chia-Ding Shih, Kirsten C. Concha-Moore, Muhanad M. Diri, Bo Hu, David Marrero, Wei Zhou, David G. Armstrong

## Notice of republication

This article was republished on April 4, 2019 to remove a Supporting Information file that was incorrectly included in the originally published article. The article’s Data Availability statement has also been updated to reflect this change. Please download this article again to view the correct version.
